# Effects of a WeChat-Based Life Review Program for Patients With Digestive System Cancer: 3-Arm Parallel Randomized Controlled Trial

**DOI:** 10.2196/36000

**Published:** 2022-08-25

**Authors:** Meihua Zheng, Xiaoling Zhang, Huimin Xiao

**Affiliations:** 1 School of Nursing Fujian Medical University Fuzhou City, Fujian Province China; 2 The Second Affiliated Hospital of Fujian Medical University Quanzhou City, Fujian Province China

**Keywords:** digestive system cancer, life review, digital technology, anxiety, depression, hope, self-transcendence, cancer, randomized controlled trial, distress, psychological, digestive system

## Abstract

**Background:**

Patients with digestive system cancer often experience psychospiritual distress. Life review is an evidence-based psychological intervention for patients with cancer, but the effects of digital life review programs are unclear, especially for patients with digestive system cancer.

**Objective:**

We examined the effects of a WeChat-based life review program on the psychospiritual well-being of patients with digestive system cancer.

**Methods:**

This study was a 3-arm parallel randomized controlled trial. Eligible patients with digestive system cancer were recruited from a university hospital in Fujian, China. They were randomized to a life review group and 2 control groups. All participants received routine care, and the life review group also received the 4-week WeChat-based life review program. Control group 1 also received a 4-week program of friendly visiting. Anxiety, depression, hope, and self-transcendence were measured at baseline and 2 days, 1 month, and 6 months after the intervention.

**Results:**

A total of 150 participants were randomly allocated to the WeChat-based life review group (n=50), control group 1 (n=50), or control group 2 (n=50). The overall dropout rate was 10% (15/150), and 92% (46/50) of participants in the the life review group completed the intervention. Significant interaction effects for time and group membership were found for anxiety (*P*<.001), depression (*P*<.001), hope (*P*<.001), and self-transcendence (*P*<.001) at all follow-up time points. For anxiety and depression, the scores did not differ significantly between the life review group and control group 1 on day 2 (*P*=.80 for anxiety, *P*=.51 for depression), but the scores were significantly lower in the life review group at month 1 and month 6 (*P*=.02 for anxiety at both months 1 and 6; *P*=.003 and *P*<.001 for depression at months 1 and 6, respectively). Significant increases in hope and self-transcendence were revealed in the life review group compared to control group participants at all follow-up sessions.

**Conclusions:**

The WeChat-based life review program was effective in reducing anxiety and depressive symptoms and in improving the level of hope and self-transcendence among patients with digestive system cancer. Though friendly visiting can also help to relieve anxiety, its effects are short-term.

**Trial Registration:**

Chinese Clinical Trial Registry ChiCTR-IOR-17011998; https://tinyurl.com/5acycpd4

## Introduction

Cancer is one of the leading causes of mortality and morbidity in the world; approximately 19 million new cases and 10 million deaths occurred in 2020, and these numbers are predicted to increase by 50% over the next 20 years. In China, digestive system cancers, including cancers of the colon, rectum, stomach, liver, and esophagus, are ranked within the top 5 diagnoses, accounting for 41% of new cancer cases and 49% of mortality [1]. A recent systematic review and meta-analysis reported a high prevalence of anxiety and depressive symptoms among patients with digestive system cancer, ranging from 50% among patients with hepatic and pancreatic cancer to 70% among patients with colorectal, esophageal, and gastric cancer [2]. Hopelessness, meaninglessness, and despair are also reported often, along with negative emotions triggered by concerns about death, seeking meaning in life, or unresolved life events associated with regret or pity [3,4].

A systematic review revealed the importance of psychological interventions in palliative care, as they can specifically address patients’ emotional difficulties and spiritual concerns [5]. Life review has been recognized as an effective psychological intervention. It is a process of recalling, evaluating, and integrating life experiences to facilitate the achievement of ego integrity [6]. Ego integrity is a state of achieving a sense of meaning and acceptance of past life events that has been found to relate to higher levels of mental health and well-being among patients in a palliative care setting [7]. Life review enables patients to express their emotions, confirm their roles in life, reassess their attitudes toward death, reorganize their perspectives toward life, and finally integrate their entire life into a more acceptable or meaningful whole [8]. Originally, life review targeted older adults’ psychosocial crises, but it has since been applied to palliative care. Accumulated evidence suggests that life review could reduce anxiety and depression, elevate hope and meaning in life, and improve self-transcendence and the quality of life of patients with cancer [9-11].

Digital technologies are increasingly being used to promote life review interventions via mobile phones, computers, wearable devices, and social media or applications [12-14]. Wise et al [15] first designed a telephone-based life review and illness narrative intervention with online resources for patients with cancer to share their personal stories and establish social networks. Afterwards, Wise et al [16] further demonstrated the effectiveness of life review in increasing feelings of peace and decreasing negative mood in patients with stage III or IV cancer after 4 months of the program. However, telephone-based life review interviews did not provide the opportunity to observe nonverbal cues, such as patients’ facial expressions and body language. Additionally, Wise reported a high dropout rate in a sample that was predominantly White, female, and had high income and high education. Recently, Dang et al [17] tested an avatar-facilitated life review intervention to reconstruct the self and identity of patients with cancer through performativity. Patients were given full-body movement devices that captured their motions and synchronized their voices onto an avatar in a virtual environment. Although the virtual environment induced a sense of immersion during the therapeutic interaction and, therefore, enhanced patients’ engagement and self-expression, the program was expensive, and there were hardware limitations [17-19].

Social media sites, such as Facebook, Instagram, and YouTube, and mobile applications have also been used to conduct life review interventions, because they enable patients to share photos, videos, and life stories [20]. WeChat is a social media platform with high popularity in 200 countries, especially China, due to its simplicity, convenience, efficiency, and mobility [21]. It allows users to interact asynchronously with each other through text messaging, voice messaging, video conferencing, and other means, as well as obtain information and resources from various WeChat platforms at any time. In 2018, our research team developed a WeChat-based life review program for patients with cancer, consisting of e-life review interviews, memory prompts, review extraction, mind space, and e-legacy products [22]. A preliminary study found that the program was acceptable, feasible, and promising in improving the psychospiritual well-being of patients with cancer [23]. Thus, this study aimed to robustly evaluate the effectiveness of the WeChat-based life review program in improving the psychospiritual well-being of patients with digestive system cancer using a 3-arm parallel randomized controlled trial.

## Methods

### Study Design and Setting

A randomized, controlled, single-blinded, 3-group pretest and repeated posttest experimental trial was conducted at the oncology department of a university-affiliated general hospital in Fujian, Southeast China. This study was performed in accordance with the CONSORT-EHEALTH checklist (Multimedia Appendix 1) [24] and was registered with the Chinese Clinical Trial Registry (ChiCTR-IOR-17011998).

### Participants

Participants were recruited from June 2019 to October 2020, with follow-up ending in April 2021. Inclusion criteria were as follows: (1) diagnosis of digestive system cancer, (2) age ≥ 18 years, (3) awareness of diagnosis and treatment, (4) ability to use WeChat, and (5) no cognitive or verbal communication impairments. The exclusion criteria included (1) current use of anxiolytics or antidepressants, (2) participation in other psychotherapeutic programs, and (3) severe disability or diagnosis with a rapid-deterioration disease (Karnofsky performance status <40%).

#### Sample Size

Power analysis was used to estimate the sample size. Assuming a power of 90%, a 2-tailed test, and an effect size of 0.33 for anxiety and 0.43 for depression, 38 and 24 participants were needed to detect changes in anxiety and depression scores, respectively [25]. For hope (effect size 0.68) and self-transcendence (effect size 0.39), sample sizes of 10 and 28 participants were needed, respectively [26]. Anticipating a 20% attrition rate, we aimed to recruit 46 participants for each study group. A final total of 50 participants was recruited for each group.

#### Randomization and Blinding

A research assistant who was not involved in subject recruitment, data collection, or the interventions conducted the randomization schedule. A research randomizer website [27] was used to generate 150 nonrepeating random number sequences. The numbers ranged from 1 to 150, with 1 to 50, 51 to 100, and 101 to 150 corresponding to the life review group, control group 1, and control group 2, respectively. Each number was separately packaged in a sequentially sealed, opaque envelope to ensure allocation concealment. In this study, the recruited participants and facilitator (the first author) were not blinded to the group assignment; another research assistant, who was blinded to group allocation, conducted the data collection and analysis.

### Interventions

All participants received routine care from medical staff at the oncology department. In addition, participants in the life review group received the 4-week WeChat-based life review program and those in control group 1 received the 4-week friendly visiting program.

#### Life Review Group

The life review group received the WeChat-based life review program along with routine care. The program consisted of a synchronous e-life review interview and asynchronous communication modules (Multimedia Appendix 2). The e-life review consisted of an individual, online, 40-to-60-minute nurse interview on WeChat, including 4 sections: present life (cancer experience); adulthood; childhood and adolescence; and summary of life. The asynchronous communication involved 4 modules. “Memory prompts” presented a set of images, music, videos, and audio-picture books relevant to each life section to trigger the participants’ memories and facilitate the life review process. “Review extraction” was a summary of meaningful events in which participants could view or leave comments. “Mind space” enabled participants to express emotions, hand down wishes, or reveal their true feelings to anyone who was important at that stage. “E-legacy product” was a digital booklet reflecting participants’ significant experiences, which they could transfer to their offspring.

The WeChat-based life review program was conducted weekly and facilitated by the first author, a registered nurse with more than 25 years of experience in clinical cancer care and 50 hours of life review training. Before the intervention, participants in the life review group installed WeChat and created a personal account. They accessed the memory prompts module to obtain an overview of the current session. Then, an e-life review interview was arranged by means of a video call with additional use of instant texts, voice messages, and emoticons. During the life review process, the facilitator monitored participants’ physical condition, emotional status, and responses to the guiding questions. Participants were also encouraged to access the asynchronous communication modules, which were available 24 hours a day, to freely review their interview content, express feelings and blessings, and provide important pictures and e-legacy products.

#### Control Group 1

Control group 1 received 4 sessions on an individual basis that provided social contact by engaging participants in daily conversation without reviewing the past. For consistency with the life review group, the 4 friendly visiting sessions were conducted by the first author through WeChat. Each visit lasted about 40 minutes, depending on the participants’ preference.

#### Control Group 2

Control group 2 received routine care, including drug treatment, nutritional support, symptom management, health education, and functional exercise.

### Measures

A self-designed questionnaire by the first author was used to collect participants’ sociodemographic information and clinical characteristics. Sociodemographic data included age, gender, marital status, education, monthly income, and religion. Clinical characteristics included the specific diagnosis; the presence or absence of chronic disease and metastasis; the use or nonuse of surgery, chemotherapy, targeted therapy, radiotherapy, and immunotherapy; and Karnofsky performance status. Karnofsky performance status was used to evaluate participants’ physical function on an 11-point scale, with 0 indicating death and a score of less than 40% indicating severe disability and rapidly progressing disease. This study only included participants with a score higher than 40%.

Psychological outcomes included anxiety and depression symptoms; these were measured by the Hospital Anxiety and Depression Scale [28]. This is a 14-item scale divided into anxiety and depressive subscales, with each of 7 items rated on a 4-point Likert scale (higher scores represent increased anxiety or depression). The Chinese version of this scale has good sensitivity and specificity [29].

Spiritual outcomes consisted of hope and self-transcendence. The 12-item Herth Hope Scale [30] was used to assess participants’ hope on a 4-point Likert scale (range 12-48). Higher scores indicate higher levels of hope. The scale has been extensively used for assessment of hope in Chinese patients with cancer; it has a Cronbach α of .87 and a construct validity of .85, indicating good reliability and validity [31]. The 15-item self-transcendence scale assessed participants’ self-transcendence [32]. Each item was rated from 1 (“not at all”) to 4 (“almost always”), with the total score ranging from 15 to 60 and a higher score indicating a higher level of self-transcendence. The Chinese version of the scale has shown good reliability (Cronbach α=.83-.87) [33].

### Data Collection and Analysis

A trained research assistant who was blinded to group assignments conducted all data collection. Outcome data were collected at baseline (T0) and 2 days (T1), 1 month (T2), and 6 months (T3) after the intervention. Statistical analysis was performed using R for Windows (version 3.5; R Foundation for Statistical Computing), with statistical significance set at *P*<.05. Normally distributed continuous variables were expressed as the mean and SD, nonnormally distributed continuous variables were presented as the median and range, and categorical variables were expressed as numbers (percentages). The Little test was used to check whether the missing data were missing completely at random. An intention-to-treat analysis was employed. Hypothesis testing used the chi-square test, the Mann-Whitney *U* test, the Fisher exact test, or a 1-way ANOVA to compare baseline data among groups. Since hierarchical linear models have more flexible data requirements and account for individual changes relative to group differences [34], they were employed for repeated measures. Both the baseline scores (intercepts) and change in scores (linear slopes) for each outcome within the groups were estimated in this model [35]. Time was represented as a dummy-coded variable to compare the outcomes at T1 to T0, T2 to T0, and T3 to T0. The effect of life review was examined based on the 3 dummy-coded time variables and the interaction effects between groups.

### Ethical Considerations

This study was approved by the Ethics Committee of Fujian Medical University (2016/00020) and the study hospital. All participants were provided with detailed information about the study, and written informed consent was obtained from each participant prior to data collection. Importantly, the data collected were kept confidential and anonymous and were used exclusively for this research.

## Results

### Participant Recruitment and Retention

**Figure 1:**
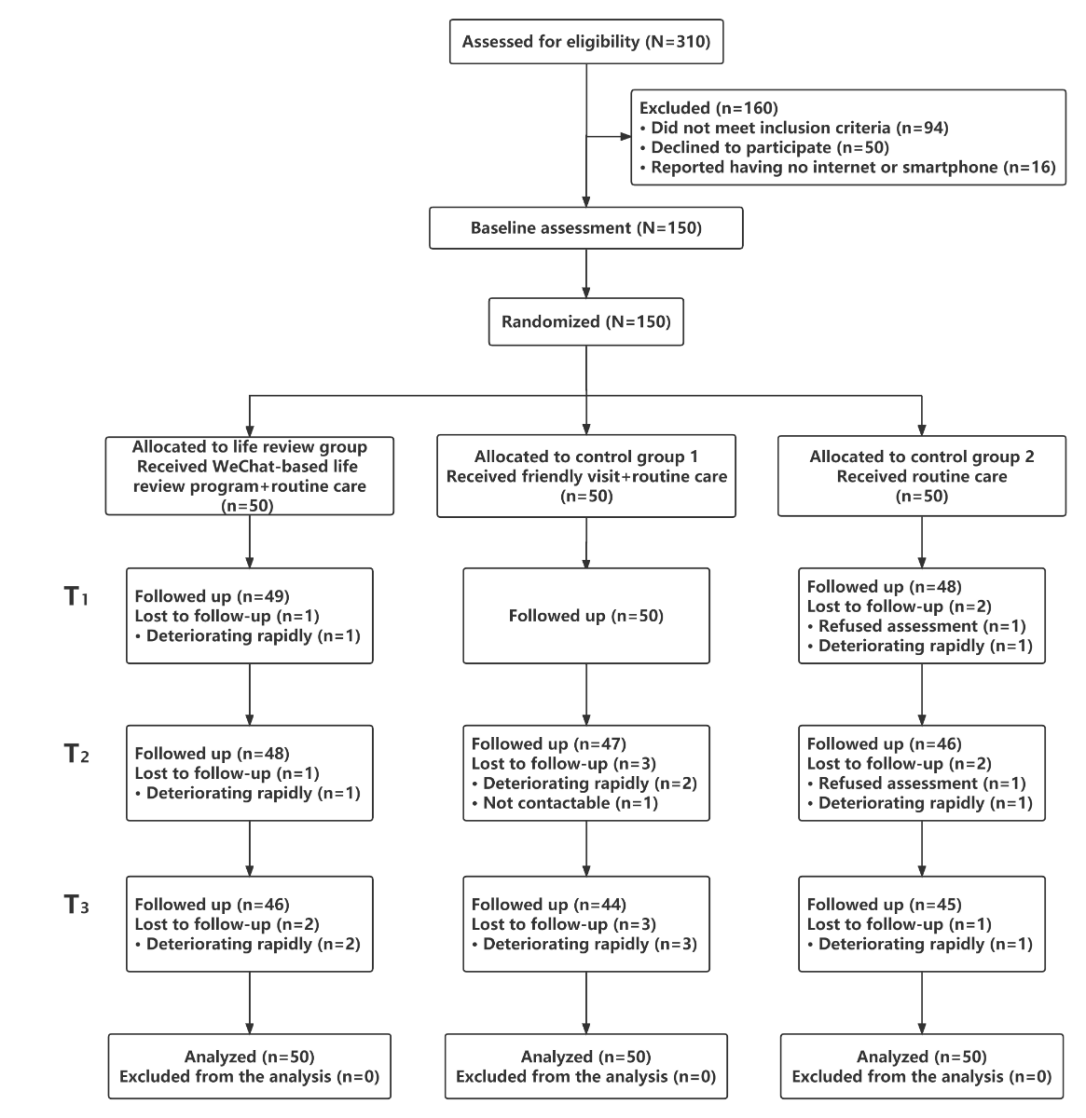
Flowchart of the study.

During the period of this study, 310 patients with digestive system cancer were assessed for eligibility; only 150 patients met the inclusion criteria and consented to participate in this study. They were randomly allocated to 3 groups: the life review group (n=50), control group 1 (n=50), and control group 2 (n=50). Fifteen participants withdrew from the study because their disease progressed (n=12), they refused to participate (n=2), or because they could not be contacted (n=1). Four of these participants were from the life review group, 6 from control group 1, and 5 from control group 2. A final total of 135 participants completed the intervention and measurements. A flowchart of the study is shown in Figure 1.

### Participant Characteristics

Table 1 shows the participants’ sociodemographic characteristics, clinical characteristics, and the baseline outcome variables across the study groups. The participants’ mean age was 58.48 (SD 9.96) years, and the majority were male (123/150, 82%), married (144/150, 96%), and affiliated with a religion (118/150, 78.7%). Less than half of the participants (74/150, 49.3%) had a primary school education level or lower and 57/150 (38%) had an average monthly household income per capita of RMB 1000 (US $148) or less. Among these participants, cancer in the digestive tract (114/150, 76%) was more common than cancer in the digestive glands (36/150, 24%); 48/150 (32%) patients had metastasis. Most patients had undergone surgery (124/150, 82.7%) or chemotherapy (102/150, 68%), and the average Karnofsky performance status was more than 60% (99/150, 66%). The groups’ demographic and clinical details were broadly comparable between the 3 groups. Interestingly, baseline anxiety and depression scores were up to 12% higher in control groups 1 and 2, but this difference could have arisen by chance alone (*P*=.89 and *P*=.17, respectively). Furthermore, this small difference would only have biased the overall estimate of effectiveness toward the null hypothesis, as it would have plausibly been slightly easier to reduce depression and anxiety scores in the control groups, as they started with a higher baseline.

**Table 1:** Baseline characteristics of participants.

Variables			Total (N=150)		Life review (n=50)		Control 1 (n=50)		Control 2 (n=50)		*F*/*χ*^2^ (*df*)			*P* value	
Age (years), mean (SD)			58.48 (9.96)		57.48 (9.29)		59.50 (10.67)		58.46 (9.96)		0.512			.60^a^	
**Gender, n (%)**												1.084 (2)			.58^b^
	Male	123 (82)		43 (35)^c^		41 (33)^c^		39 (38)^c^							
	Female	27 (18)		7 (26)^c^		9 (33)^c^		11 (41)^c^							
**Marital status, n (%)**												1.261 (1)			.32^d^
	Married	144 (96)		48 (96）		48 (96)		48 (96)							
	Unmarried/ widowed/ divorced/ separated	6 (4）		2 (4）		2 (4)		2 (4)							
**Monthly household income (US $), (n %)**												6.128 (6)			.42^b^
	≤15	57 (38)		19 (33)^c^		20 (35)^c^		18 (32)^c^							
	15-44	33 (22)		8 (24)^c^		12 (36)^c^		13 (39)^c^							
	44-88	36 (24)		12 (33)^c^		9 (25)^c^		15 (42)^c^							
	>88	24 (16)		11 (46)^c^		9 (38)^c^		4 (17)^c^							
**Religion, n (%)**												1.986 (2)			.37^b^
	Yes	118 (78.7)		41 (82）		36 (72）		41 (82）							
	No	32 (21.3)		9 (18）		14 (28）		9 (18）							
**Education level, n (%)**												7.494 (6)			.28^b^
	Primary school or below	74 (49.3）		25 (34)^c^		23 (31)^c^		26 (35)^c^							
	Junior middle school	45 (30）		11 (24)^c^		15 (33)^c^		19 (42)^c^							
	Senior high school	21 (14）		10 (47)^c^		7 (33)^c^		4 (19)^c^							
	Tertiary or above	10 (6.7）		4 (40)^c^		5 (50)^c^		1 (10)^c^							
**Chronic disease, n (%)**												0.273 (2)			.87^b^
	Yes	40 (26.7)		12 (24)		14 (28)		14 (28)							
	No	110 (73.3)		38 (76)		36 (72)		36 (72)							
**Diagnosis, n (%)**												2.851 (2)			.24^b^
	Cancer in digestive tract	114 (76)		35 (70)		42 (84)		37 (74)							
	Cancer in digestive glands	36 (24)		15 (30)		8 (16)		13 (26)							
**Metastasis, n (%)**												2.206 (2)			.33^b^
	Yes	48 (32)		18 (36)		18 (36)		12 (24)							
	No	102 (68)		32 (64)		32 (64)		38 (76)							
**Surgery, n (%)**												4.001 (2)			.14^b^
	Yes	124 (82.7)		37 (74)		44 (88)		43 (86)							
	No	26 (17.3)		13 (26)		6 (12)		7 (14)							
**Chemotherapy, n (%)**												2.206 (2)			.33^b^
	Yes	102 (68)		32 (64)		38 (76)		32 (64)							
	No	48 (32)		18 (36)		12 (24)		18 (36)							
**Targeted therapy, n (%)**												2.542 (2)			.37^d^
	Yes	6 (4)		1 (17)		4 (67)		1 (17)							
	No	144 (96)		49 (34)		46 (32)		49 (34)							
**Radiotherapy, n (%)**												2.990 (2)			.22^b^
	Yes	21 (14)		7 (14)		10 (20)		4 (8)							
	No	129 (86)		43 (86)		40 (80)		46 (92)							
**Immunotherapy, n (%)**												0.398 (2)			>.99^d^
	Yes	9 (6）		3 (6)		3 (6)		3 (6)							
	No	141 (94）		47 (94）		47 (94)		47 (94)							
**Karnofsky performance status, n (%)**												2.317 (2)			.35^b^
	≤60	51 (34)		13 (26)		18 (35)		20 (39)							
	>60	99 (66)		37 (37)		32 (32)		30 (60)							
**Baseline outcome scores, mean (SD)**															
	Anxiety	3.81 (3.72)		3.74 (3.92)		3.68 (3.38)		4.02 (3.90)		0.118			.89^a^		
	Depression	4.13 (3.83)		3.60 (3.53)		5.06 (4.30)		4.02 (3.97)		1.817			.17^a^		
	Hope	36.57 (3.80)		36.76 (4.53)		36.50 (3.64)		36.46 (3.17)		0.091			.91^a^		
	Self-transcendence	45.97 (6.28)		46.40 (6.46)		46.02 (6.02)		45.44 (6.39)		0.295			.75^a^		

^a^Calculated with ANOVA.

^b^Calculated with the *Χ*^2^ test.

^c^The denominator used to calculate these percentages is the value for n in the “Total” column of the same row.

^d^Calculated with the Fisher exact test.

### Effects on Outcome Variables

Table 2 shows the mean (SD) for the outcome variables at baseline and at the 3 follow-up sessions. A hierarchical linear model was employed to examine the change in outcome variables at each time point (Table 3). Overall, the interaction effects of the intervention on anxiety, depression, hope, and self-transcendence between groups over time were statistically significant.

**Figure 2:**
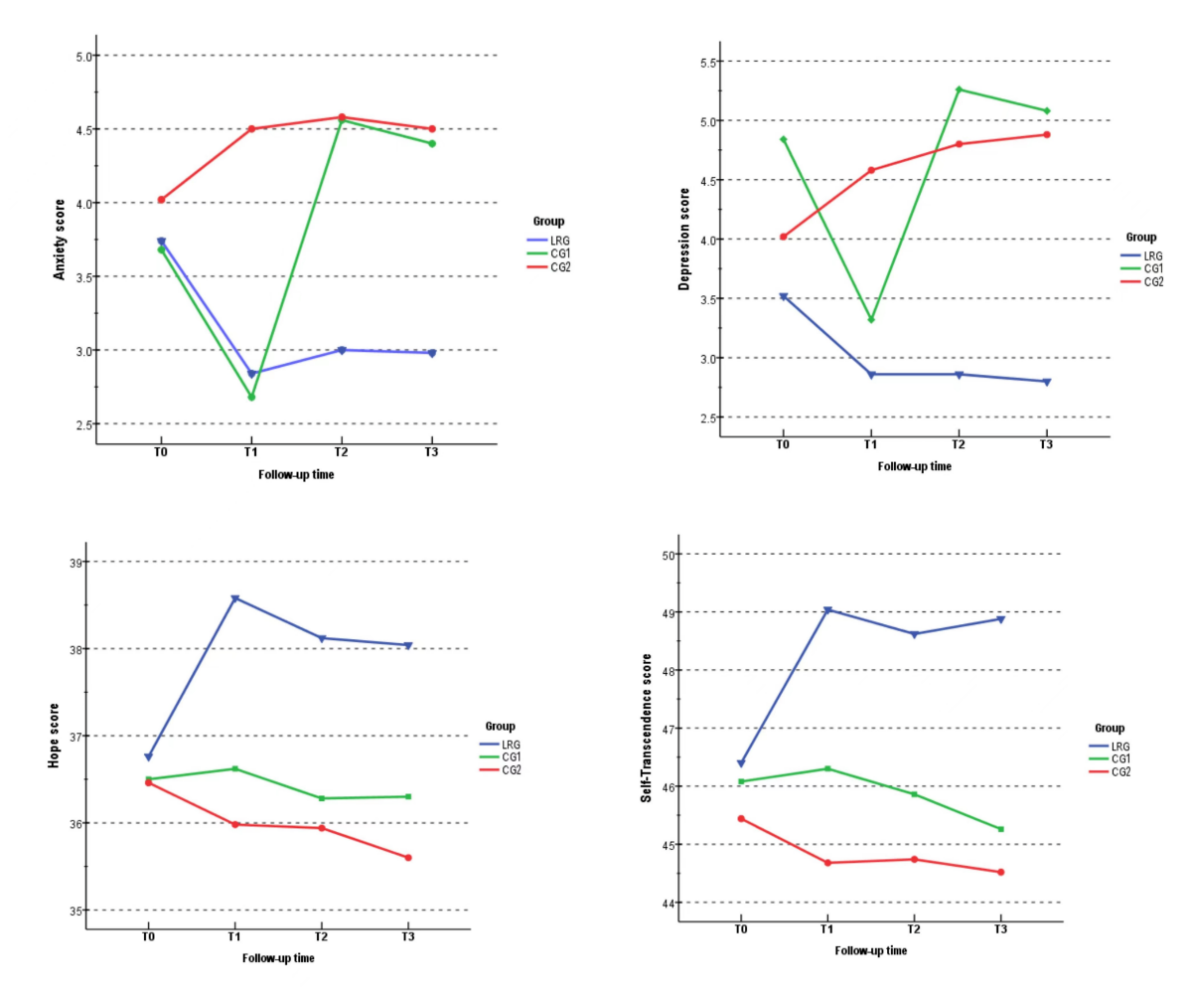
Changes in the mean scores for anxiety, depression, hope, and self-transcendence in the patients over time. CG1: control group 1; CG2: control group 2; LRG: life review group; T0: baseline; T1: 2 days postintervention; T2: 1 month postintervention; T3: 6 months postintervention.

Figure 2 shows the change over time in the mean (SD) scores for anxiety, depression, hope, and self-transcendence. Specific comparisons of outcome variables between groups at each time point and within groups are presented in Multimedia Appendix 3 and Multimedia Appendix 4. For anxiety, there was a significant decrease in the life review group at T1, T2, and T3 compared to baseline (*P*<.001, *P*<.001, and *P*=.002, respectively), indicating that the scores remained stable after the intervention. In the control groups, the anxiety score tended to show an overall upward trend, except for a decrease from baseline to T1 in control group 1. No significant difference in anxiety score was found between participants in the life review group and control group 1 at T1 (*P*=.80). However, the anxiety score was significantly lower in the life review group than in control group 1 at T2 and T3 (*P*=.02 for both). Compared with control group 2, the scores significantly decreased in the life review group at all follow-up sessions (*P*=.01, *P*=.02, and *P*=.01 at T1, T2, and T3, respectively).

A similar tendency was found in the depression score. There was a significant decrease in the life review group at all periods, and an increase in control group 2 from baseline (*P*=.02, *P*<.001, and *P*=.002 for T1, T2, and T3, respectively). As for control group 1, depression decreased significantly at T1 (*P*<.001) and increased at T2 and T3 (*P*=.07 and *P*=.37, respectively). The depression scores did not differ significantly between the life review group and control group 1 at T1 (*P*=.51), but depression was significantly lower in the life review group than in control group 1 at T2 and T3 (*P*=.003 and *P*<.001, respectively). There was also a significant difference in the depression score between the life review group and control group 2 at all follow-up sessions (*P*=.02 for both T1 and T2, *P*=.004 for T3).

A significant difference was observed in the hope score between the life review group and the 2 control groups at all follow-up sessions. Intragroup comparisons showed a significant increase in hope in the life review group after the intervention at T1, T2, and T3 (all *P*<.001). No significant differences were found over time in control group 1 (*P*=.55, *P*=.32, and *P*=.46 for T1, T2, and T3, respectively), while significant decreases were found in control group 2 at T1, T2, and T3 (*P*=.02 for both T1 and T2, *P*=.002 for T3).

In terms of self-transcendence, there was a significant difference between the life review group and the 2 control groups at T1, T2, and T3. Intragroup comparisons showed a significant increase in self-transcendence in the life review group after the intervention at T1, T2, and T3 (all *P*<.001). No statistically significant differences were found in self-transcendence over time for control group 1 (*P*=.46, *P*=.51, and *P*=.24 for T1, T2, and T3, respectively), while significant decreases were found in control group 2 at T1, T2, and T3 (*P*=.01, *P*=.04, *P*=.01 for T1, T2, and T3, respectively).

**Table 2:** Outcome variables at baseline and posttests (N=150; n=50 in each group).

Outcome variables		T0,^a^ mean (SD)	T1,^b^ mean (SD)	T2,^c^ mean (SD)	T3,^d^ mean (SD)
**Anxiety**					
	Life review	3.74 (3.92)	2.84 (2.61)	3.00 (3.18)	2.98 (2.71)
	Control 1	3.68 (3.39)	2.68 (2.55)	4.56 (2.43)	4.40 (2.37)
	Control 2	4.02 (3.90)	4.50 (4.06)	4.58 (4.22)	4.50 (3.54)
**Depression**					
	Life review	3.52 (3.40)	2.86 (2.80)	2.86 (2.87)	2.80 (1.91)
	Control 1	4.84 (4.04)	3.32 (3.05)	5.26 (4.36)	5.08 (3.62)
	Control 2	4.02 (3.97)	4.58 (4.39)	4.80 (4.66)	4.88 (4.51)
**Hope**					
	Life review	36.76 (4.53)	38.58 (4.12)	38.12 (3.75)	38.04 (3.58)
	Control 1	36.50 (3.64)	36.62 (3.49)	36.28 (3.31)	36.30 (3.54)
	Control 2	36.46 (3.17)	35.98 (3.25)	35.94 (3.40)	35.60 (3.05)
**Self-transcendence**					
	Life review	46.40 (6.46)	49.04 (5.80)	48.62 (6.03)	48.88 (5.44)
	Control 1	46.08 (6.06)	46.30 (5.36)	45.86 (5.84)	45.26 (5.43)
	Control 2	45.44 ( 6.39	44.68 (6.27)	44.74 (6.25)	44.52 (5.95)

^a^T0: baseline.

^b^T1: 2 days postintervention.

^c^T2: 1 month postintervention.

^d^T3: 6 months postintervention.

**Table 3:** Parameter estimates of the models with random intercept and random slope.

Model (random intercept and slope; fixed effects)	Anxiety					Depression					Hope					Self-transcendence			
	Estimate	Standard error	*t* test (*df*)	*P* value	Estimate		Standard error	*t* test (*df*)	*P* value	Estimate		Standard error	*t* test (*df*)	*P* value	Estimate		Standard error	*t* test (*df*)	*P* value
Intercept	4.020	0.468	8.599 (170.11)	<.001	4.020		0.526	7.642 (168.22)	<.001	36.460		0.508	71.817 (168.78)	<.001	45.440		0.842	53.992 (162.09)	<.001
LRG^a^	–0.280	0.661	–0.424 (170.11)	.67	–0.500		0.744	–0.672 (168.22)	.50	0.300		0.718	0.418 (168.78)	.68	0.960		1.190	0.807 (162.09)	.42
CG1^b^	–0.340	0.661	–0.514 (170.11)	.61	0.820		0.744	1.102 (168.22)	.27	0.040		0.718	0.056 (168.78)	.96	0.640		1.190	0.538 (162.09)	.59
T1^c^	0.480	0.204	2.354 (441)	.02	0.560		0.221	2.538 (441)	.01	–0.480		0.216	–2.227 (441)	.03	–0.760		0.301	–2.522 (441)	.01
T2^d^	0.560	0.204	2.747 (441)	<.001	0.780		0.221	3.535 (441)	<.001	–0.520		0.216	–2.413 (441)	.02	–0.700		0.301	–2.323 (441)	.02
T3^e^	0.480	0.204	2.354 (441)	.02	0.860		0.221	3.898 (441)	<.001	–0.860		0.216	–3.990 (441)	<.001	–0.920		0.301	–3.053 (441)	<.001
LRG:T1	–1.380	0.288	–4.786 (441)	<.001	–1.220		0.312	–3.910 (441)	<.001	2.300		0.305	7.546 (441)	<.001	3.400		0.426	7.977 (441)	<.001
CG1:T1	–1.480	0.288	–5.133 (441)	<.001	–2.080		0.312	–6.666 (441)	<.001	0.600		0.305	1.969 (441)	.05	0.980		0.426	2.299 (441)	.02
LRG:T2	–1.300	0.288	–4.509 (441)	<.001	–1.440		0.312	–4.615 (441)	<.001	1.880		0.305	6.168 (441)	<.001	2.920		0.426	6.851 (441)	<.001
CG1:T2	0.320	0.288	1.110 (441)	.27	–0.360		0.312	–1.154 (441)	.25	0.300		0.305	0.984 (441)	.33	0.480		0.426	1.126 (441)	.26
LRG:T3	–1.240	0.288	–4.301 (441)	<.001	–1.580		0.312	–5.064 (441)	<.001	2.140		0.305	7.021 (441)	<.001	3.400		0.426	7.977 (441)	<.001
CG1:T3	0.240	0.288	0.832 (441)	.41	–0.620		0.312	–1.987 (441)	.05	0.660		0.305	2.165 (441)	.03	0.100		0.426	0.235 (441)	.82
Random intercept variance	9.889	3.145	12.619	3.552	N/A^f^		N/A	N/A	N/A	11.726		3.424	N/A	N/A	33.145		5.757	N/A	N/A
Residual variance	1.039	1.019	1.217	1.103	N/A		N/A	N/A	N/A	1.161		1.078	N/A	N/A	2.271		1.507	N/A	N/A

^a^LRG: life review group.

^b^CG1: control group 1.

^c^T1: 2 days postintervention.

^d^T2: 1 month postintervention.

^e^T3: 6 months postintervention.

^f^N/A: not applicable.

## Discussion

### Primary Findings

This is the first study to evaluate the effects of an online life review intervention on the psychospiritual well-being of patients with digestive system cancer; this study adopted a rigorous randomized controlled trial design with a very large sample size and long follow-up time. Our results show that the WeChat-based life review program could reduce anxiety and depressive symptoms and improve feelings of hope and self-transcendence among patients with digestive system cancer for a period of at least 6 months after the intervention. Our findings also revealed that friendly visiting might reduce anxiety symptoms in the short term; however, it did not improve depressive symptoms, feelings of hope, or self-transcendence among patients with digestive system cancer.

### Participants’ Adherence

Fifteen of 150 participants (10%) withdrew after 6 months of follow-up, which is an attrition rate lower than that of previous online life review studies [16,36]. Specifically, in the life review group, 46 out of 50 patients completed the whole program, indicating that the WeChat-based life review program was well implemented. A possible reason may be that life review is a naturally occurring, universal mental process among patients with cancer in the final life stage [37]. Patients with deteriorating health or low functionality can still participate in life review, even when lying in bed [38]. The low dropout rate may also be due to the simplicity, convenience, efficiency, and mobility of the WeChat-based program, especially among patients with cancer [39]. Considering the time and space limitations, it provided a range of ways to communicate, including text and pictures, voice messages, and video calls, allowing patients to participate in the program at any convenient time and location.

### Patients’ Outcomes

Among patients with digestive system cancer, anxiety symptoms significantly decreased from baseline up to 6 months after the life review intervention, which is consistent with previous research findings [40]. It is also worth noting that friendly visiting might help reduce anxiety symptoms in the short term, but not the long term. Scholars have put forward the idea that expression is an effective way of regulating emotions, allowing patients to feel supported by others, sort out their thoughts, and release negative emotions [41,42]. In this study, both friendly visiting and life review were conducted in a virtual, individual session where patients could feel safe and comfortable and reveal their innermost feelings in a familiar environment. Friendly visiting allowed patients to express their complaints about the disease and helped them divert their attention to other achievable things, leading to a temporary decrease in anxiety. Conversely, the WeChat life review program’s long-term effectiveness may be due to opportunities for patients to retrieve positive thoughts, express and re-evaluate negative emotions, focus on the balance of positive and negative reminiscences, and integrate memories into a meaningful whole. Though painful memories may be picked up during the life review process, the facilitator offers guidance to consider these memories from other perspectives. Accordingly, patients are able to let go, accept, or even gain fresh insights into their lives, finally achieving self-integrity [8]. Meanwhile, the friendly visiting intervention focused on daily conversations without reviewing the past and with no guidance from the facilitator; thus, its effect on anxiety was unstable, with patients’ anxiety scores going back to baseline or increasing 1 and 6 months after the intervention.

Our study results further confirmed the long-term benefits of the life review intervention for patients with digestive system cancer. The WeChat-based life review program significantly decreased depressive symptoms long-term, for at least 6 months after the intervention. This is in line with the findings of Lamers et al [43], who reported positive effects 6 months after the implementation of a similar program to ours among adults with moderate depressive symptoms.

The decrease in depression scores might be due to the accumulation of positive thoughts [44]. The WeChat-based life review program facilitated the retrieval of happy feelings from positive memories, prompted by means of appreciating images, music, videos, and audio-picture books. It also provided an opportunity for patients to learn from the past and affirm their contributions to families and society, which may induce positive emotions. On the other hand, reconciling negative experiences contributes to relieving depressive symptoms and improving one’s emotional state [45]. In the process of life review, patients were encouraged to optimistically interpret the negative experiences in their own way to give positive meaning to the unpleasant stories, difficulties, and disappointments in their lives. From different perspectives, those negative experiences were reconstructed to bring about desired changes in the patients’ views of themselves and their world. Finally, various life experiences were integrated into an acceptable whole and the patients moved toward acceptance of their lives.

Significant improvements in hope were also perceived among patients with cancer who participated in the WeChat-based life review program, consistent with previous research [11,46]. It might be that life review helps patients collate and learn from their pasts and reaffirm their contributions and accomplishments to their families and society, strengthening their awareness of their existence. During the life review process, patients may also perceive support from the facilitator and their family, since positive correlations have been observed between social support and increased hope [47]. Alternatively, patients could have set goals that matched their ability, making them more likely to be successful, thus increasing their feelings of hope. Through the life review intervention, patients become systematically aware of their life trajectory, gain a better understanding of their current situation, and take action congruent with their palliative situation. In addition, the e-legacy products may be beneficial to increase the patients’ hope. A systematic review has reported that patients are in a positive state when reviewing their lives, especially when they view, touch, and appreciate the e-legacy product made in the life review process [8]; such feelings are maintained for a period of time.

Significant increases in self-transcendence were observed among patients with digestive system cancer who participated in the WeChat-based life review program, which is consistent with previous findings [23,48]. According to Reed [49], self-transcendence is an expansion of one’s conceptual boundaries; inwardly, through introspective activities, outwardly, through concerns about others’ welfare, and temporally, by integrating perceptions of one’s past and future to enhance the present. The following reasons explain how our WeChat-based life review program could improve self-transcendence. First, during life review, patients recall and evaluate life experiences, and they are encouraged to express their feelings, reorganize their perspectives, and reconstruct the meaning of their lives, which can strengthen the inward domain of self-transcendence. Second, the WeChat-based life review program helps patients connect with their surroundings, which can improve outward transcendence by engaging in reciprocal relationships. While reviewing their lives, patients can reconsider and reflect on their connections to family and society, thereby discovering important emotional support around them. Third, the WeChat-based life review program integrates patients’ past and future to improve their present, which is helpful in enhancing the temporal domain of self-transcendence. In sum, the WeChat-based life review program enabled patients to gradually focus on caring for others, transcending the present and achieving self-transcendence by introspection and gaining a harmonious view of the past, present, and future.

This study explored new possibilities for psychological interventions in oncology. The program took advantage of WeChat’s increased availability and scalability to life review interventions, which is expected to overcome the obstacles of geographic distance and traffic issues, benefiting more patients, especially remotely located individuals. WeChat also promises to reduce personnel resources for delivery compared with face-to-face interventions. We recommend that future studies also examine the cost-effectiveness of this program, which could convince facilitators to engage in practice and integrate this intervention into transitional care in the community for patients with cancer.

### Limitations

The limitations of this study should be noted. First, the WeChat-based life review program may not be suitable for people with poor literacy skills, because they may encounter difficulties in completing the 4 life review modules. Second, participant recruitment took place in only 1 hospital; hence, the generalizability of the findings may be limited. Multicenter and transregional research with a rigorous design is recommended in future research. In addition, this study covered multiple types of cancer diagnoses; future studies may consider selecting patients with the same cancer diagnosis.

### Conclusions

Our WeChat-based life review program showed short- and long-term effectiveness in reducing anxiety and depressive symptoms and improving hope and self-transcendence among patients with digestive system cancer. By contrast, friendly visiting might reduce anxiety symptoms, but did not influence depression, hope, or self-transcendence. Accordingly, this WeChat-based life review program should be integrated into transitional care for digestive system cancer.

## Appendix

Multimedia Appendix 1 CONSORT-eHEALTH checklist (V 1.6.1).

Multimedia Appendix 2 Screenshot of life review.

Multimedia Appendix 3 Comparison of four outcome variables between groups at each time point.

Multimedia Appendix 4 Comparison of four outcome variables within groups.

## Abbreviations

T0: baseline
T1: 2 days postintervention
T2: 1 month postintervention
T3: 6 months postintervention
