# Supplementary figures and images for: Effects of a WeChat-Based Life Review Program for Patients With Digestive System Cancer: 3-Arm Parallel Randomized Controlled Trial
Source: J Med Internet Res. 2022 Aug 25;24(8):e36000. doi: 10.2196/36000 (PMC9459832; doi:10.2196/36000)

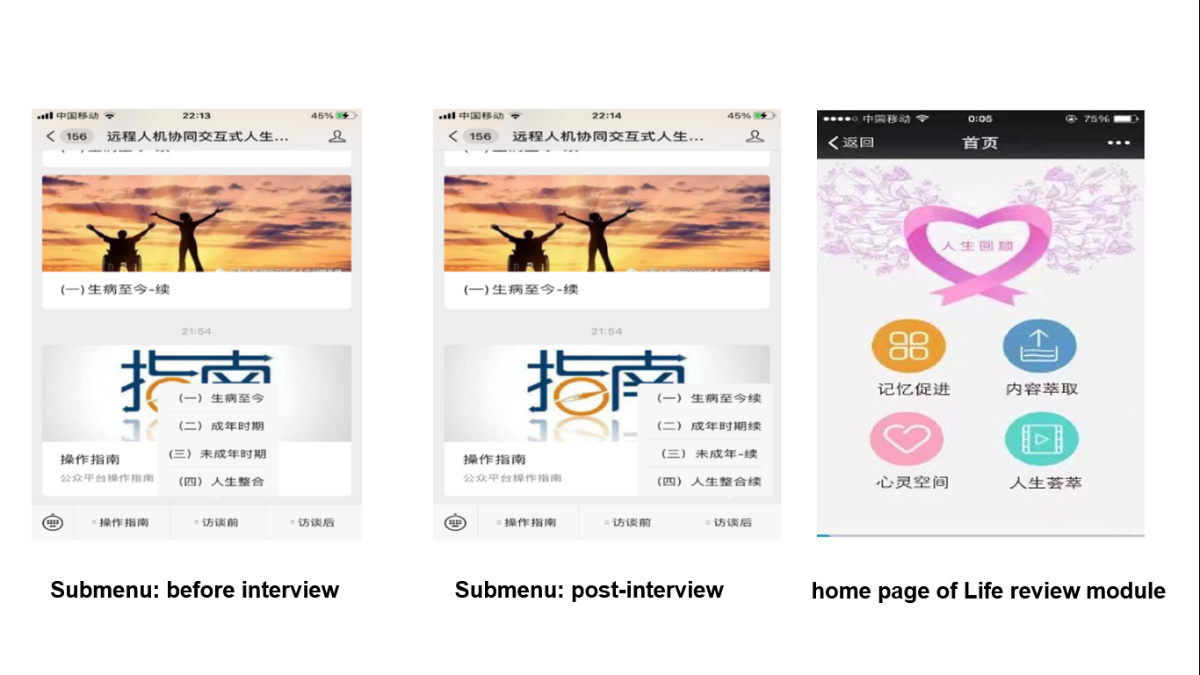

Supplement: Multimedia Appendix 2 [file jmir_v24i8e36000_app2.png]
