# Supplementary material for: Effects of a WeChat-Based Life Review Program for Patients With Digestive System Cancer: 3-Arm Parallel Randomized Controlled Trial
Source: J Med Internet Res. 2022 Aug 25;24(8):e36000. doi: 10.2196/36000 (PMC9459832; doi:10.2196/36000)
Supplement: Multimedia Appendix 3 [file jmir_v24i8e36000_app3.doc]

**Multimedia Appendix 3** **Comparison of four outcome variables between groups**

| Variables | Group | T0  *P* (95%CI) | T1  *P* (95%CI) | T2  *P* (95%CI) | T3  *P* (95%CI) |
| --- | --- | --- | --- | --- | --- |
| Anxiety | LRG vs. CG1 | .94  -1,419 to 1.539 | .80  -1.086 to 1.406 | .02**  -2.888 to -0.232 | .02***  -2.571 to -0.269 |
|  | LRG vs. CG2 | .71  -1.759 to 1.199 | .01***  -2.906 to -0.414 | .02**  -2.908 to -0.252 | .01***  -2.671 to 0.369 |
|  | CG1 vs. CG2 | .66  -1.819 to 1.139 | .004***  -3.066 to -0.574 | .98  -1.348 to -1.308 | .86  -1.251 to 1.051 |
| Depression | LRG vs. CG1 | .09  -2.827 to 0.187 | .51  -0.917 to -2.637 | .003**  -3.996 to -0.804 | <.001*  -3.670 to -0.890 |
|  | LRG vs. CG2 | .51  -2.007 to 1.007 | .02**  -3.097 to -0.343 | .02**  -3.536 to -0.344 | .004***  -3.470 to -0.690 |
|  | CG1 vs. CG2 | .28  -0.687 to 2.327 | .07  -0.117 to 2.637 | .57  -1.136 to 2.056 | .78  -1.190 to 1.590 |
| Hope | LRG vs. CG1 | .73  -1.249 to 1.769 | .01***  0.522 to 3.398 | .01***  0.460 to 3.220 | .01***  0.397 to 3.083 |
|  | LRG vs. CG2 | .70  -1.209 to 1.809 | <.001*  1.162 to 4.038 | .002***  0.800 to 3.560 | .001*  1.097 to 3.783 |
|  | CG1 vs. CG2 | .96  -1.469 to 1.549 | .38  -0.798 to 2.078 | .63  -1.040 to 1.720 | .31  -0.643 to 2.043 |
| Self-transcendence | LRG vs. CG1 | .80  -2.173 to 2.813 | .02**  0.440 to 5.040 | .02**  0.372 to 5.148 | .002**  -5.839 to -1.401 |
|  | LRG vs. CG2 | .45  -1.533 to 3.453 | <.001*  2.060 to 6.660 | .002***  1.492 to 6.268 | .001*  2.141 to 6.579 |
|  | CG1 vs. CG2 | .61  -1.853 to 3.133 | .17  -3.920 to 0.680 | .36  -3.508 to 1.268 | .51  -1.479 to 2.959 |

**at each time point**

LRG= Life Review Group; CG1= Control Group 1; CG2= Control Group 2.
