# Supplementary material for: Effects of a WeChat-Based Life Review Program for Patients With Digestive System Cancer: 3-Arm Parallel Randomized Controlled Trial
Source: J Med Internet Res. 2022 Aug 25;24(8):e36000. doi: 10.2196/36000 (PMC9459832; doi:10.2196/36000)
Supplement: Multimedia Appendix 4 [file jmir_v24i8e36000_app4.doc]

**Multimedia Appendix 4 Comparison of four outcome variables within groups**

| Variables | Group (n) | T0 vs T1  *P* (95%CI) | T0 vs T2  *P* (95%CI) | T0 vs T3  *P* (95%CI) |
| --- | --- | --- | --- | --- |
| Anxiety | LRG (n=50) | <.001*  0.436 to 1.364 | <.001*  0.323 to 1.157 | .002**  0.277 to 1.243 |
|  | CG1 (n=50) | <.001*  0.536 to 1.464 | <.001*  -1.297 to -o.463 | .004***  -1.203 to -0.237 |
|  | CG2 (n=50) | .04  -0.944 to -0.16 | .01***  -0.977 to -0.143 | .05  -0.963 to 0.003 |
| Depression | LRG (n=50) | .01***  0.207 to 1.113 | .01***  0.199 to 1.121 | .01***  0.191 to 1.249 |
|  | CG1 (n=50) | <.001*  1.067 to 1.973 | .07  -0.881 to 0.041 | .37  -0.769 to 0.289 |
|  | CG2 (n=50) | .02***  -1.013 to -0.107 | <.001*  -1.241 to -0.319 | .002**  -1.389 to -0.331 |
| Hope | LRG (n=50) | <.001*  -2.213 to -1.427 | <.001*  -1.798 to -0.922 | <.001*  -1.814 to -0.746 |
|  | CG1 (n=50) | .55  -0.513 to 0.273 | .32  -0.218 to 0.658 | .46  -0.334 to 0.734 |
|  | CG2 (n=50) | .02***  0.087 to 0.873 | .02**  0.082 to 0.958 | .002***  0.326 to 1.394 |
| Self-transcendence | LRG (n=50) | <.001*  -3.220 to -2.060 | <.001*  -2.883 to -1.557 | <.001*  -3.190 to -1.770 |
|  | CG1 (n=50) | .46  -0.800 to 0.360 | .51  -0.443 to 0.883 | .24  0.110 to 1.530 |
|  | CG2 (n=50) | .01***  0.180 to1.340 | .04  0.037 to 1.363 | .01***  0.210 to 1.630 |

LRG= Life Review Group; CG1= Control Group 1; CG2= Control Group 2.
